# Rate variation in parasitic plants: correlated and uncorrelated patterns among plastid genes of different function

**DOI:** 10.1186/1471-2148-5-16

**Published:** 2005-02-15

**Authors:** Nelson D Young, Claude W dePamphilis

**Affiliations:** 1 Department of Biomedical Sciences, Tufts University School of Veterinary Medicine, 200 Westboro Road, North Grafton, MA 01536 USA; 2 Department of Biology and Huck Institutes for the Life Sciences, Pennsylvania State University, University Park, PA 16802 USA

## Abstract

**Background:**

The analysis of synonymous and nonsynonymous rates of DNA change can help in the choice among competing explanations for rate variation, such as differences in constraint, mutation rate, or the strength of genetic drift. Nonphotosynthetic plants of the Orobanchaceae have increased rates of DNA change. In this study 38 taxa of Orobanchaceae and relatives were used and 3 plastid genes were sequenced for each taxon.

**Results:**

Phylogenetic reconstructions of relative rates of sequence evolution for three plastid genes (*rbcL*, *matK *and *rps2*) show significant rate heterogeneity among lineages and among genes. Many of the non-photosynthetic plants have increases in both synonymous and nonsynonymous rates, indicating that both (1) selection is relaxed, and (2) there has been a change in the rate at which mutations are entering the population in these species. However, rate increases are not always immediate upon loss of photosynthesis. Overall there is a poor correlation of synonymous and nonsynonymous rates. There is, however, a strong correlation of synonymous rates across the 3 genes studied and the lineage-speccific pattern for each gene is strikingly similar. This indicates that the causes of synonymous rate variation are affecting the whole plastid genome in a similar way. There is a weaker correlation across genes for nonsynonymous rates. Here the picture is more complex, as could be expected if there are many causes of variation, differing from taxon to taxon and gene to gene.

**Conclusions:**

The distinctive pattern of rate increases in Orobanchaceae has at least two causes. It is clear that there is a relaxation of constraint in many (though not all) non-photosynthetic lineages. However, there is also some force affecting synonymous sites as well. At this point, it is not possible to tell whether it is generation time, speciation rate, mutation rate, DNA repair efficiency or some combination of these factors.

## Background

Rates of DNA sequence evolution vary among taxa and among genes, and the causes of this variation are many. In some cases, generation time has been shown to be correlated with rates in plants. For example, annual plants can sometimes have higher rates of DNA evolution than perennials [1]. In one study, it was shown that long-lived woody grasses exhibit slower rates than short-lived herbaceous ones [2]. However, a more extensive set of 33 phylogenetically independent comparisons failed to find a generation time effect for plants in general [3].

A useful method for distinguishing among the potential causes of rate variation is to separately examine nonsynonymous rates (*r*_*N*_) and synonymous rates (*r*_*S*_). For example, when *r*_*N *_increases relative to *r*_*S*_, relaxation of purifying selection is a possible explanation. However, when *r*_*S *_increases, but the *r*_*N*_/*r*_*S *_ratio is not greatly affected, then an increase in the mutation rate is a possibility. An example of this is *Plantago *mitochondrial DNA [4]. However, a decrease in DNA repair efficiency could also explain such a change. In addition, population processes, such as genetic drift could play a role. Reduced effective population size (N_e_) can increase the fixation rate of neutral and slightly deleterious mutations. Thus, if slightly deleterious mutations are common, both *r*_*S *_and *r*_*N *_are expected to be higher when N_e _is low [5,6].

One expectation of this drift-based hypothesis is that *r*_*N *_will vary from protein to protein, as each protein will have different functional constraints and thus a different proportion of slightly deleterious mutations. In contrast, *r*_*S *_is expected to be similar among proteins when the cause is a change in mutation rate or repair efficiency[7].

During founder-effect speciation, genetic drift can be expected to increase the substitution rate, even at silent sites. This has been called the speciation-rate hypothesis [8]. For example, speciation rate has been used to explain the difference in non-coding DNA substitution rates between the sister genera *Utricularia *and *Pinguicula *in the plant family Lentibulariaceae [9].

Rates can also vary if the underlying mutation rate varies [10,11] or if DNA repair is impaired [12,13]. Although rates themselves are hard to measure, the number of synonymous (*d*_*S*_) and nonsynonymous (*d*_*N*_) substitutions can be measured and are used to compare rates and calculate rate ratios.

*Epifagus virginiana*, a nonphotosynthetic plant, has an increased rate of sequence evolution for plastid DNA in general [14-17] and relative rates tests of the plastid *rps2 *gene indicate a significant increase for both *d*_*N *_and *d*_*S*_. This suggests that purifying selection is at least partially relaxed and that there has been an increase in the rate at which mutations are entering the population in this species, due to increased mutation rate or lax DNA repair. *MatK*, another plastid gene, is characterized by a partial relaxation of purifying selection in the clade containing *Epifagus*, *Orobanche *and *Boschniakia *[18]. In this paper, we explore rate variation in *E. virginiana *and 38 of its relatives for three plastid genes: *rps2*, *matK*, and *rbcL*. Each of these genes is present in photosynthetic relatives of Epifagus, is accelerated (or even lost) in Epifagus or related parasitic plants. Although plastid encoded, the three genes encode proteins that participate in different processes in the plastid. *rps2 *encodes the ribosomal protein S2 in small subunit ribosome, *matK *is an intron maturase, and *rbcL *encodes the large subunit in the CO_2_-fixing enzyme RUBISCO. We ask several questions: When does the rate increase observed in *Epifagus *begin, relative to the evolutionary loss of photosynthesis? What are the causes? Relaxation of constraint? More mutations entering the population? Are these patterns consistent across multiple plastid genes?

## Results

Phylogenies of the Orobanchaceae and relatives were constructed using maximum parsimony (MP) and maximum likelihood (ML). The MP analysis discovered four most parsimonious trees of 3817 steps, with CI = 0.6275, CI (excluding uninformative characters) = 0.5118, and RC = 0.3888. The strict consensus tree was unresolved as to the position of *Schwalbea *relative to the *Alectra-Orobanche *clade, the *Bartsia-Melampyrum *clade and the *Castilleja-Pedicularis *clade. It was also unresolved concerning the relationships among the outgroups *Mimulus*, *Kigelia*, *Hemimeris*, *Verbascum*, *Antirrhinum *and *Veronica*.

**Figure 1:**
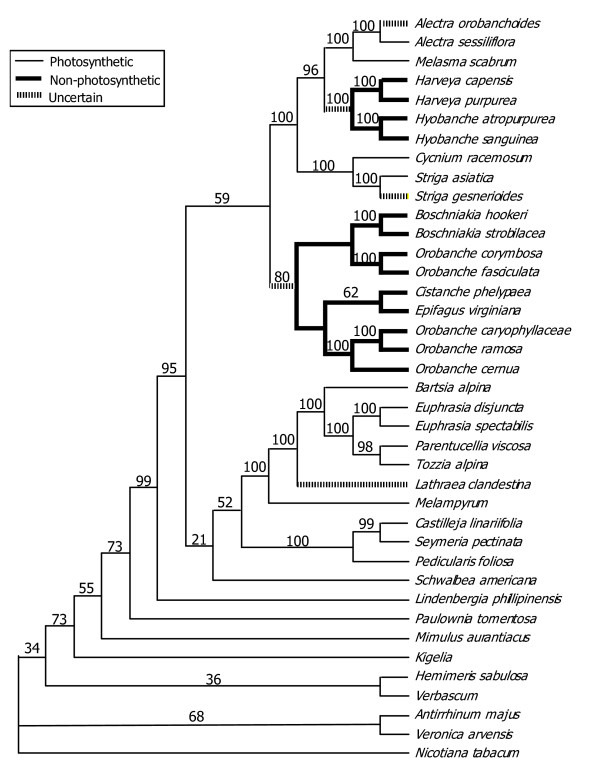
MP/ML consensus tree with MP bootstrap values

The ML analysis found two trees, with -ln likelihood values of 24525.88663. The strict consensus of these trees was unresolved, but in a different place, regarding the position of the *Cistanche-Epifagus *clade. When the MP consensus and the ML consensus were combined into a semistrict consensus tree, a completely resolved tree resulted. This tree is shown in Fig. 1.

**Figure 2:**
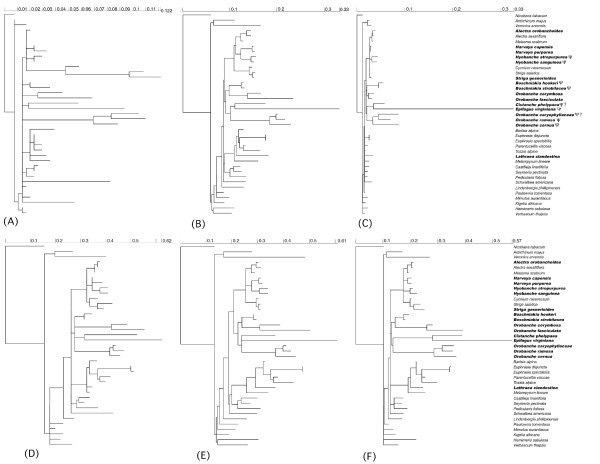
**Nonsynonymous and synonymous rates of change in three genes. **Nonphotosynthetic taxa are named in bold. (A) *rps2 *nonsynonymous branch lengths. (B) *matK *nonsynonymous branch lengths. (C) *rbcL *nonsynonymous branch lengths. (D) *rps2 *synonymous branch lengths. (E) *matK *synonymous branch lengths. (F) *rbcL *synonymous branch lengths. Taxa with *rbcL *pseudogenes are identified with Ψ. Uncertain pseudogene status is indicated by "Ψ?" [20, 26].

All three gene trees exhibited statistically significant rate heterogeneity (p < 0.0005), as assessed by the Kishino-Hasegawa (K-H) test [19]. Synonymous and nonsynonymous branch lengths for each of the three genes are shown reconstructed in Fig. 2.

**Figure 3:**
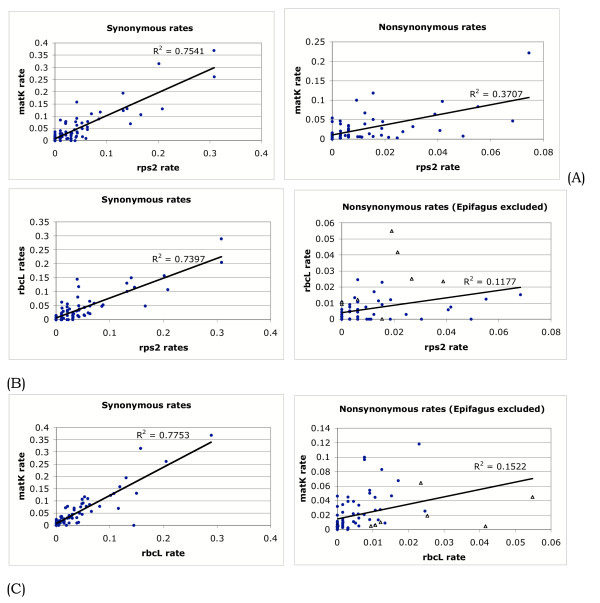
**Correlations across each possible pair of genes for synonymous and nonsynonymous rates. **(A) *rps2 *versus *matK*. (B) *rps2 *versus *rbcL*. (C) *matK *versus *rbcL*. All nonsynonymous comparisons involving *rbcL *pseudogenes are indicated with a hollow triangle.

**Figure 4:**
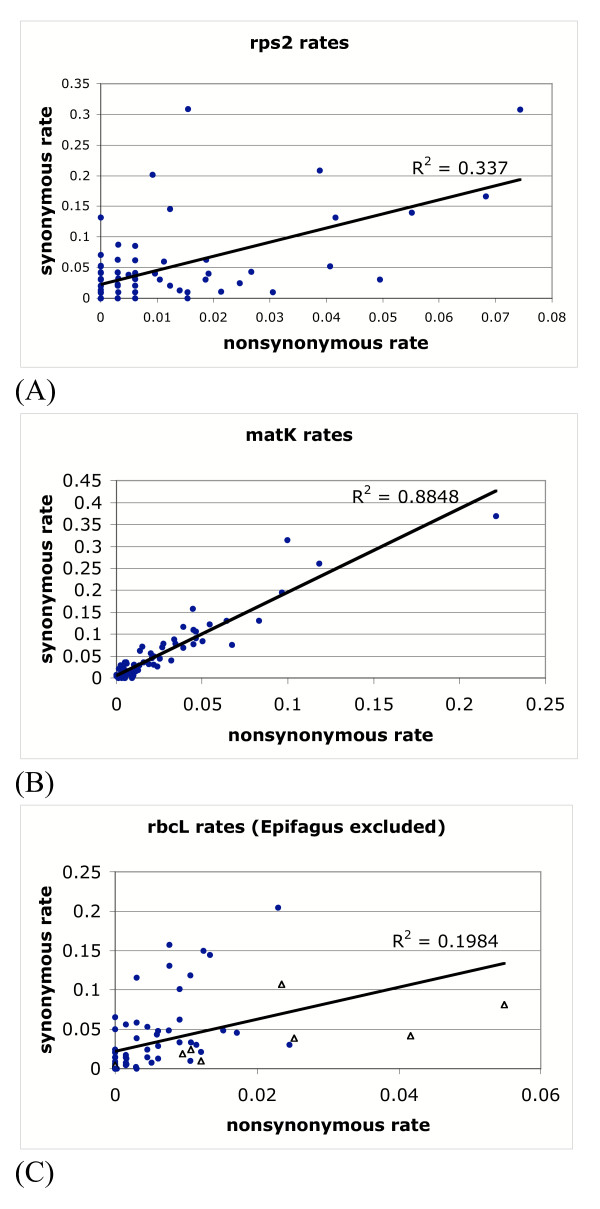
**Correlation plots of synonymous and nonsynonymous rates within each gene. **(A) *rps2 *rates. (B) *matK *rates. (C) *rbcL *rates. In 4C, the *Epifagus *pseudogene has been excluded and the other pseudogenes are indicated with a hollow triangle.

The correlation analyses show that there is a higher correlation of synonymous evolution across genes than nonsynonymous evolution (Fig. 3). They also show that within *rps2 *and *rbcL *synonymous and nonsynonymous evolution is poorly correlated, but in *matK*, the correlation is better (Fig. 4).

Some of the non-photosynthetic plants, (*Epifagus*, *Cistanche*, and the *Orobanche *species) have increases in both synonymous and nonsynonymous rates. Rates are not, however, increased in *Boschniakia*, *Harveya*, *Hyobanche*, *Lathaea*, *Alectra orobanchoides*, and *Striga gesnerioides*.

Synonymous rates vary markedly among taxa. For example, the branches leading to *Epifagus *are at least two or three times as long as those for most of the photosynthetic taxa. Despite this among-taxon variation, the pattern for each gene is strikingly similar (Fig. 2D, E, F). For example, sister taxa (e.g. *Veronica *and *Antirrhinum*, *Euphrasia *and *Panetucellia/Tozzia*, *Epifagus *and *Cistanche*, *O. fasciculata *and *O. corymbosa*) usually show an identical pattern of who is faster than whom. This indicates that the causes of synonymous rate variation are affecting the whole plastid genome in a similar way.

There is even more extensive variation in nonsynonymous rates, both among taxa and among genes. This is not surprising because these genes have different functions and some of the taxa are photosynthetic while others are not. The scale bars in Figs. 2A, B, and 2C show the overall differences in nonsynonymous rates. *MatK *is much more rapidly evolving than the other two genes for nearly all taxa, but *Epifagus' rbcL *pseudogene has a similar rate. When looking at taxa across genes, there is much less consistency than with the synonymous rates. There are some big differences, such as the branch lengths for *Striga*, *Cycnium *and *Schwalbea*, and the *Euphrasia *species. Overall the picture is more complex, as could be expected if there are many causes of variation, differing from taxon to taxon and gene to gene.

The pattern of *matK *rate variation is very similar in the synonymous and nonsynonymous figures. This fits well with the fact that it is less constrained overall, as can be seen by comparing the scale bars in Figs. 2A, B, and 2C.

Rates were compared by using two categories at a time and testing for significant differences using likelihood ratio tests. These tests are summarized in Table 2. In general, it was found that the nonphotosynthetic plants have higher synonymous and nonsynonymous rates of change, although when tested separately, it was found that *Harveya*, *Hyobanche *and *Boschniakia *do not have higher synonymous rates.

**Table 1:** Specimens used for DNA sequencing, with GenBank accession numbers for rps2, matK, and rbcL sequences.

	**GenBank accession numbers**		
	
**Species**	***rps2***	***matK***	***rbcL***
*Alectra orobanchoides*	U48741	AF489960	AF026819
*Alectra sessiliflora*	U48742	AF051977	AF026820
*Antirrhinum majus*	U48766	AF051978	L11688
*Bartsia alpina*	U48751	AY849600	AF190903
*Boschniakia hookeri*	U48757	AF051979	AF026817
*Boschniakia strobilacea*	U48758	AF051980	AF26818
*Castilleja lineariifolia*	U48739	AF051981	AF026823
*Cistanche phelypaea*	AY849597	AF056149	AY849862
*Cycnium racemosum*	U48745	AY849601	AF026826
*Epifagus virginiana*	EPFCPCG	EPFCPCG, AF051982	EPFCPCG
*Euphrasia disjuncta*	AY849598	AY849602	AY849863
*Euphrasia spectabilis*	U48752	AY849603	AY849864
*Harveya capensis*	AF055142	AF489961	AF026829
*Harveya purpurea*	U48749	AF051984	AF026830
*Hemimeris sabulosa*	U48765	AF051985	AF123668
*Hyobanche atropurpurea*	AY849599	AF051986	AF026831
*Hyobanche sanguinea*	U48750	AF051987	AF026832
*Kigelia africana*	U48764	AF051988	AF102648
*Lathraea clandestina*	U48755	AF051989	AF026833
*Lindenbergia phillipinensis*	AF055151	AF051990	AF123664
*Melampyrum sylvaticum*	AF055148	AF051991	
*Melampyrum lineare*			AF026834
*Melasma scabrum*	U48743	AY849604	AF190904
*Mimulus aurantiacus*	AF055154	AY849605	AF026835
*Nicotiana tabacum*	Z00044	Z00044	Z00044
*Orobanche caryophyllacea*	AF055145	AF051992	AY582187
*Orobanche cernua*	AF055147	AF056147	U73968
*Orobanche corymbosa*	U48760	AF051993	U73969
*Orobanche fasciculata*	AF055143	AF051994	U73970
*Orobanche hederae*	AF055146	AF051995	AF078682
*Orobanche ramosa*	U48761	AF056148	U73971
*Parentucellia viscosa*	U48753	AY849606	AY849865
*Paulownia tomentosa*	AF055255	AF051997	L36447
*Pedicularis foliosa*	U48740	AF489959	AF026836
*Schwalbea americana*	AF055150	AF051998	AY849866
*Seymeria pectinata*	AF055141	AF051999	AF026837
*Striga asiatica*	U48746	AF052000	AF026838
*Striga gesnerioides*	U48747	AF489963	AF026839
*Tozzia alpina*	U48754	AF052001	AF026843
*Verbascum blattaria*	U48763		
*Verbascum thapsus*		AF052002	L36452
*Veronica arvensis*	U48768	AF052003	
*Veronica persica*			L36453

Taxonomic authorities, localities and voucher information can be found in [25], with the exception of *Euphrasia disjuncta *Fernald & Wiegand, *Hyobanche atropurpurea *Bolus., and *Veronica persica *Poir.

Purifying selection is relaxed in the nonphotosynthetic plants for all three genes. The test values are as follows. *matK*: 2×LR = 10.67, df = 1, p = 0.001; *rbcL*: 2×LR = 31.3, df = 1, p = 2.2 × 10^-8^; r*ps2*: 2×LR = 8.56, df = 1, p = 0.00343.

Another way to describe the difference in the pattern of synonymous and non-synonymous rates is to say that the former are more correlated across genes. This can be seen in Fig. 3, which shows plots comparing two genes at a time. In comparisons including rbcL nonsynonymous rates, the data point from the *Epifagus *pseudogene has been excluded. Its unconstrained evolution is not typical of "nonsynonymous" change and its position on the plot made it an extreme outlier with an enormous influence on the regression line.

For *rps2 *and *rbcL*, the synonymous plots are more highly correlated, whereas for *matK*, which is relatively unconstrained, they are about the same.

## Discussion

The dramatic rate increase observed in *Epifagus *[14], with branches 5–10 times as long as other taxa, can now be seen to have begun earlier in the history of the Orobanchaceae. It is shown to be composed of increases in both synonymous and nonsynomymous rates. The general pattern is that many of the non-photosynthetic plants, such as *Epifagus*, *Cistanche*, and the *Orobanche *species, have increases in both synonymous and nonsynonymous rates, indicating that both (1) selection is relaxed, and (2) there has been a change in the rate at which mutations are entering the population in these species. However, rate increases are not immediate upon loss of photosynthesis, since we do not see increases in *Boschniakia*, *Harveya*, *Hyobanche*, *Lathaea*, *Alectra orobanchoides*, and *Striga gesnerioides*. This pattern is similar to that found using smaller data sets [15,20]. Separate analyses of synonymous and nonsynonymous rates give us some insight into potential mechanisms.

The speciation rate hypothesis predicts that more speciose clades should have a faster *r*_*s *_(and therefore larger *d*_s_) than a species-poor sister group. This was suggested as a cause of rate variation in non-coding DNA in the Lentibulareaceae [9]. In this study, not all genera have been sampled, and those that have are often represented by one or two species. In addition, some genera may not be monophyletic (for example, *Orobanche *is not). Thus, accurate numbers of species cannot be assigned to individual branches or clades. However, a few things can be noted. *Euphrasia*, with ~170 spp., is clearly more speciose than its sister group, with 3 spp. It has a somewhat faster *d*_s_. However, the *Schwalbea *lineage, with a single species, has a fairly high *d*_s_. Its position is not certain, but its sister group is probably the *Bartsia *– *Melampyrum *clade (>300 spp.), the *Castilleja *– *Pedicularis *clade, (>700 spp.), or the union of the two. These groups have do not have dramatically higher *d*_s _values; in fact the *Castilleja *– *Pedicularis *clade's value is slightly lower.

Differences in generation time may play some role in the observed rate variation. However, as was found previously [15], the pattern is not clear. Since most of the genera sampled in this study contain both annuals and perennials, it is likely that most branches on the tree actually represent a combination of annual and perennial evolutionary history. However, there are some intriguing details that might merit further study. The clade containing *Bartsia*, *Euphrasia *and *Melampyrum *contains mostly annuals [21] and has some high *d*_s _values, as one would expect from a generation time effect. However, both *Euphrasia *and *Melampyrum *contain almost exclusively annuals and have very different rates. Likewise, the large clade containing *Boschniakia *and *Epifagus *contains mostly annuals and has an overall high *d*_s_. The perennials *Boschniakia *and *Cistanche *have lower *d*_s _than their sister taxa, which also supports the generation time hypothesis, but there is as much variation among categories (perennial, annual) as between categories.

## Conclusions

The distinctive pattern of rate increases in Orobanchaceae has at least two causes. It is clear that there is a relaxation of constraint in many (though not all) non-photosynthetic lineages. However, there is also some force affecting synonymous sites as well. At this point, it is not possible to tell whether it is generation time, speciation rate, mutation rate, DNA repair efficiency or some combination of these factors. Clearly, generating additional data from nuclear and mitochondrial genes would help us to more clearly distinguish among these hypotheses. Some of the above-mentioned hypotheses (generation-time, speciation rate) would be expected to affect nuclear and mitochondrial genomes in a similar fashion, whereas factors affecting mutation rate or efficiency of DNA repair would not, as these process involve different, though perhaps overlapping, sets of enzymes in each of the three genomes [22-24].

## Methods

### Sampling

We sampled 15 photosynthetic and 16 nonphotosynthetic Orobanchaceae, and eight outgroup taxa. The specimens used and their GenBank accession numbers are given in Table 1.

**Table 2:** P-values of the likelihood ratio tests.

	*rps2*			*matK*			*rbcL*		
	*r*_*N*_	*r*_*S*_	ratio (ω)	*r*_*N*_	*r*_*S*_	ratio (ω)	*r*_*N*_	*r*_*S*_	ratio (ω)

photo. vs nonphoto.	1.4 × 10^-11^	0.0014	0.0034	0	0.077 (NS)	0.001	0	2.3 × 10^-8^	2.2 × 10^-8^
photo. vs OEC	0	6.3 × 10^-10^	7.2 × 10^-8^	0	7.6 × 10^-14^	0.0038	0	0	2.7 × 10^-11^
photo. vs HHB	0.41 (NS)	0.0066	0.24 (NS)	1.4 × 10^-6^	4.5 × 10^-9^	0.23 (NS)	0.076 (NS)	0.0011	8.1 × 10^-5^

The two categories at left were compared for nonsynonymous rate differences (*r*_*N*_), synonymous rate differences (*r*_*S*_) or rate ratio (ω) differences. "Photo." refers to photosynthetic branches, "nonphoto." refers to nonphotosynthetic branches, "OEC" refers to *Orobanche*, *Epifagus *and *Cistanche *branches, and "HHB" refers to *Harveya*, *Hyobanche *and *Boschniakia *branches.

### Amplification and sequencing

We amplified and sequenced *rps2 *as in [15], *matK *as in [25], and *rbcL *as in [26]. A total of 15 new sequences were generated for this study, including 3 *rps2*, 7 *matK*, and 5 *rbcL *sequences.

### Alignment

The *rps2 *alignment was simple, containing only two small indels. For *matK *and *rbcL*, a search for the best alignment was conducted using Clustal X and a variety of alignment parameters. Alignments were evaluated according to the following optimality criterion: whichever alignment yields the MP tree(s) with the highest consistency is considered the best alignment[27]. For alignment assessment, MP analyses with and without indel characters were used. When used, indel characters were generated with the program GapCoder [28](available from ), which uses the simple indel coding method of Simmons and Ochoterena [29]. The rescaled consistency (RC) index [30] of the resulting parsimony analyses was used to assess alignment optimality, with one exception: very low gap opening penalties (GOP), such as 3 or less were excluded. These low GOP values lead to inflated RC values, due to the large numbers of gaps, which reduce the treelength and the homoplasy. *rps2 *had just one small indel and was aligned by eye. For *matK*, the optimal computer alignment was generated using GOP = 5 and gap extension penalty (GEP) = 1. Transitions were weighed the same as transitions. The RC from the analysis with indel characters included was 0.3693. The RC without indel characters was 0.3759. The alignment was then adjusted by eye. This final alignment yielded RC values of 0.3878 (indel characters included) and 0.3763 (indel characters excluded).

For *rbcL*, the optimal computer alignment was generated using GOP = 5 GEP = 3. Transitions were weighed the same as transitions. The RC from the analysis with indel characters included was 0.4679. The RC without indel characters was 0.4437. The alignment was then adjusted by eye. This final alignment yielded RC values of 0.4635 (indel characters included) and 0.4499 (indel characters excluded).

### Phylogenetic analyses

For *rps2*, positions homologous to positions 48–660 of the *Nicotiana *gene were used. For *matK*, the entire gene was used. For *rbcL*, positions homologous to *Nicotiana *gene positions 5–1325 were used. The three genes were then combined into a single data set. PAUP* 4.0b8 [31] was used to conduct a MP heuristic search, including indel characters, and using *Nicotiana tabacum *as the outgroup taxon, TBR branch swapping and 100 random addition replicates. Bootstrap analyses were conducted with the same settings, except with only 40 random addition orders. 500 bootstrap replicates were performed.

ML analyses excluded indels. Using the hLRT method of the program ModelTest 3.06 [32], the ML model of GTR+G was selected as the best evolutionary model for the combined data set. Base frequencies (A = .298, C = .177, G = .203) and substitution rates (A-C = 1.51, A-G = 2.11, A-T = 0.264, C-G = 0.788, C-T = 2.62, G-T = 1) were obtained from the MP trees. Among-site variation was included in the model, based on a gamma distribution with four categories. A heuristic search was conducted, similar to the MP search, but with only 10 random addition replicates, each limited to the examination of 5000 rearrangements.

#### Rates of DNA change

Overall rate heterogeneity was assessed using the K-H test as implemented in PAUP, using the same ML analyses, except that the starting tree was a neighbor-joining tree and the analysis was limited to 40 rearrangements. Nonsysnonymous and synonymous changes were reconstructed on branches using the codon-based likelihood model of Muse and Gaut [33], as implemented in HYPHY for MacOS, ver. 0.95 beta [[34] 2004], available at . At least nine of the rbcL "genes" are probably pseudogenes. These are indicated in Figure 2C. Seven of these have already been discussed elsewhere [20]. The *Orobanche caryophyllaceae *and *Cistanche phelypaea *"genes" have internal stop codons and thus are probably also pseudogenes. There may also be other pseudogenes with intact ORFs, making their pseudogene status less obvious [35]. Once a pseudogene is formed, it is no longer constrained for a protein function, so synonymous and nonsynonymous changes can no longer be formally defined. Moreover, changes that would have been synonymous and nonsynonymous are now expected at equal rates. Thus, by including these sequences in the tests, we get additional evidence that constraint is relaxed in nonphotosynthetic plants. Therefore, even for the pseudogenes, we have still calculated the synonymous and nonsynonymous rates separately, assuming a reading frame based on alignment to the other genes in the data set, and have indicated the pseudogenes in Fig. 2C.

Rate increases were compared among categories of taxa (such as photosynthetic and non-photosynthetic), using *d*_*N*_, *d*_*S*_, and the *d*_*N*_/*d*_*S *_ratio (ω) in likelihood ratio tests [36]. These tests were conducted using HYPHY and the category assignments of the branches are those shown in Figure 1. In addition, a previous study [15] indicated that some nonphotosynthetic branches might not have rate increases. Thus, two subsets of the nonphotosynthetic branches were tested: (1) *Orobanche*, *Epifagus *and *Cistanche *branches and (2) *Harveya*, *Hyobanche *and *Boschniakia *branches. Each of these tests used the data set from a single gene and compared two nested hypotheses: H_1_: the photosynthetic and non-photosynthetic branches share a single value (for one of the parameters *d*_*N*_, *d*_*S *_or ω). H_2_: the photosynthetic and non-photosynthetic branches have two separate values. If the tree has a significantly higher likelihood under H_2_, that is taken as evidence that the nonphotosynthetic branches have higher rates. Scatter plots and correlation tests were used to examine the degree of correlation between synonymous and nonsynonymous sites within a gene, and also to see if either class of sites was correlated between genes.

## Authors' contributions

NDY and CWD conceived of and designed the study together. NDY did the sequencing, data analyses and drafted the manuscript. CWD provided the genomic DNA samples and provided the conducive laboratory environment, both physical and intellectual, as well as many suggestions for the manuscript.
